# Supplementary material for: Effect of Apple Juice Enrichment with Selected Plant Materials: Focus on Bioactive Compounds and Antioxidant Activity
Source: Foods. 2022 Dec 25;12(1):105. doi: 10.3390/foods12010105 (PMC9818660; doi:10.3390/foods12010105)
Supplement: Supplementary file 1 [file foods-12-00105-s001.zip › foods-2101609-supplementary.pdf]

Supplementary File

# Effect of Apple Juice Enrichment with Selected Plant Materials: Focus on Bioactive Compounds and Antioxidant Activity

**Katarzyna Angelika Gil, Aneta Wojdyło, Paulina Nowicka, Paola Montoro, and Carlo Ignazio Giovanni Tuberoso**

**Table S1.** Spearman correlation coefficients and significance level ($p \leq 0.05$ and $p \leq 0.01$, see legend at the bottom of the table).

| | L* | a* | b* | DM[1] | Ashes | TSS[2] | TA[3] | TSS[2]/TA[3] | pH | Vitamin C | Fructose | Sorbitol | Glucose | Sucrose | Total sugars |
|---|---|---|---|---|---|---|---|---|---|---|---|---|---|---|---|
| a* | -0.2063 | | | | | | | | | | | | | | |
| b* | 0.9567 | 0.0324 | | | | | | | | | | | | | |
| DM[1] | -0.1847 | 0.7826 | 0.0424 | | | | | | | | | | | | |
| Ashes | -0.5372 | 0.3853 | -0.3666 | 0.7654 | | | | | | | | | | | |
| TSS[2] | -0.2300 | 0.8895 | 0.0100 | 0.9795 | 0.6894 | | | | | | | | | | |
| TA[3] | -0.0928 | 0.8830 | 0.1536 | 0.9617 | 0.6055 | 0.9791 | | | | | | | | | |
| TSS[2]/TA[3] | -0.5070 | 0.3221 | -0.3320 | 0.4641 | 0.6744 | 0.4598 | 0.3465 | | | | | | | | |
| pH | -0.5726 | 0.4268 | -0.4483 | 0.7601 | 0.8651 | 0.7002 | 0.6516 | 0.3220 | | | | | | | |
| Vitamin C | 0.4205 | 0.7632 | 0.5984 | 0.5339 | -0.0759 | 0.6206 | 0.6818 | 0.0689 | -0.0933 | | | | | | |
| Fructose | -0.2752 | 0.7402 | -0.1659 | 0.6974 | 0.4634 | 0.7456 | 0.6833 | 0.0335 | 0.6100 | 0.4089 | | | | | |
| Sorbitol | 0.1211 | 0.7195 | 0.2157 | 0.6067 | 0.1750 | 0.6625 | 0.6536 | -0.1802 | 0.3218 | 0.6526 | 0.9118 | | | | |
| Glucose | -0.5635 | 0.8817 | -0.3837 | 0.6980 | 0.4642 | 0.8079 | 0.7222 | 0.4770 | 0.5234 | 0.4943 | 0.7376 | 0.5799 | | | |
| Sucrose | 0.3509 | 0.6335 | 0.5795 | 0.8223 | 0.4588 | 0.7850 | 0.8519 | 0.2942 | 0.3533 | 0.7334 | 0.3788 | 0.4993 | 0.3272 | | |
| Total sugars | -0.3383 | 0.8280 | -0.1983 | 0.7543 | 0.4948 | 0.8186 | 0.7512 | 0.1561 | 0.6196 | 0.4784 | 0.9844 | 0.8829 | 0.8417 | 0.4212 | |
| Oxalic acid | -0.3971 | 0.2137 | -0.2710 | 0.7358 | 0.9299 | 0.6144 | 0.5569 | 0.5761 | 0.8591 | -0.1245 | 0.3107 | 0.0636 | 0.3051 | 0.4761 | 0.3357 |
| Citric acid | -0.4173 | 0.2423 | -0.2814 | 0.7477 | 0.9427 | 0.6322 | 0.5745 | 0.5955 | 0.8667 | -0.1129 | 0.3210 | 0.0677 | 0.3295 | 0.4838 | 0.3501 |
| Tartaric | 0.4562 | -0.4357 | 0.2604 | -0.2556 | -0.2635 | -0.3389 | -0.3366 | -0.6634 | -0.1400 | -0.1992 | 0.1680 | 0.3100 | -0.4726 | -0.1784 | 0.0077 |
| Malic acid | -0.4280 | 0.9264 | -0.2074 | 0.8721 | 0.5816 | 0.9410 | 0.9092 | 0.4124 | 0.6870 | 0.5578 | 0.7503 | 0.6175 | 0.9291 | 0.5711 | 0.8434 |
| Quinic acid | 0.2434 | 0.8759 | 0.4388 | 0.5720 | 0.0026 | 0.6860 | 0.7269 | 0.0890 | 0.0094 | 0.9668 | 0.5600 | 0.7398 | 0.6340 | 0.6723 | 0.6288 |
| Shikimic acid | -0.7421 | 0.2783 | -0.6441 | 0.6401 | 0.8906 | 0.5789 | 0.4626 | 0.6343 | 0.8801 | -0.2325 | 0.4114 | 0.0629 | 0.5394 | 0.1785 | 0.4606 |

| | | | | | | | | | | | | | | | |
|---|---|---|---|---|---|---|---|---|---|---|---|---|---|---|---|
| Total organic acids | -0.1967 | 0.7911 | 0.0313 | 0.9993 | 0.7619 | 0.9828 | 0.9604 | 0.4846 | 0.7502 | 0.5430 | 0.6964 | 0.6043 | 0.7163 | 0.8163 | 0.7579 |
| Sugar/organic acids | -0.3064 | 0.2543 | -0.3789 | -0.0693 | -0.1243 | 0.0351 | -0.0454 | -0.3571 | 0.0810 | -0.0083 | 0.6593 | 0.5927 | 0.3773 | -0.3751 | 0.5969 |
| TP[4] | -0.1336 | 0.1113 | -0.1657 | 0.4895 | 0.5123 | 0.3933 | 0.3380 | -0.1535 | 0.6897 | -0.1157 | 0.6828 | 0.5662 | 0.1840 | 0.2010 | 0.5880 |
| CUPRAC | 0.0189 | -0.1489 | -0.0665 | 0.2628 | 0.2772 | 0.1408 | 0.1658 | -0.4403 | 0.5953 | -0.2755 | 0.3539 | 0.2847 | -0.1287 | 0.0814 | 0.2462 |
| FRAP | -0.2869 | 0.1583 | -0.2827 | 0.6065 | 0.6212 | 0.5025 | 0.4584 | 0.0216 | 0.8430 | -0.1290 | 0.5593 | 0.3812 | 0.2899 | 0.2583 | 0.5187 |
| ORAC | -0.6689 | 0.0815 | -0.6553 | 0.3950 | 0.7593 | 0.3254 | 0.2228 | 0.2014 | 0.8425 | -0.4837 | 0.5302 | 0.1879 | 0.2843 | -0.0664 | 0.4798 |
| DPPH• | -0.1806 | -0.0668 | -0.2141 | 0.4465 | 0.5150 | 0.3114 | 0.3030 | -0.0852 | 0.7572 | -0.2735 | 0.3114 | 0.1574 | 0.0426 | 0.1822 | 0.2588 |
| ABTS•+ | -0.1988 | 0.0673 | -0.2105 | 0.5558 | 0.5887 | 0.4332 | 0.4027 | -0.0387 | 0.8032 | -0.1703 | 0.4926 | 0.3380 | 0.1703 | 0.2587 | 0.4365 |
| Total anthocyanins | -0.8219 | 0.2854 | -0.8446 | 0.0102 | 0.1147 | 0.1286 | -0.0116 | 0.2116 | 0.2540 | -0.1734 | 0.4055 | 0.1460 | 0.6532 | -0.4972 | 0.4595 |
| Total hydroxybenzoic acids | -0.0828 | 0.7913 | 0.1409 | 0.9737 | 0.6548 | 0.9646 | 0.9480 | 0.4925 | 0.6204 | 0.6563 | 0.6317 | 0.6000 | 0.7061 | 0.8539 | 0.7071 |
| Total hydroxycinnamic acids | 0.1596 | 0.7637 | 0.2984 | 0.3263 | -0.2720 | 0.4773 | 0.5461 | -0.1104 | -0.1035 | 0.8486 | 0.3758 | 0.5504 | 0.5877 | 0.3893 | 0.4608 |
| Dihydrochalcones | -0.1248 | 0.2374 | -0.1257 | -0.3746 | -0.5336 | -0.2010 | -0.1552 | -0.3773 | -0.3464 | 0.1376 | 0.0449 | 0.0899 | 0.1784 | -0.3840 | 0.0654 |
| Total flavan-3-ols | -0.6137 | 0.5208 | -0.5986 | 0.1765 | 0.0120 | 0.3206 | 0.2160 | 0.1184 | 0.2134 | 0.1873 | 0.5488 | 0.4018 | 0.7936 | -0.2557 | 0.6184 |
| Polymeric proanthocyanidins | 0.2051 | 0.0819 | 0.1769 | 0.5042 | 0.3769 | 0.3880 | 0.3710 | -0.2003 | 0.5061 | 0.0980 | 0.5655 | 0.5867 | 0.0530 | 0.4003 | 0.4729 |
| Total flavonols | -0.7466 | 0.2848 | -0.6814 | -0.0442 | 0.2065 | 0.0680 | 0.0109 | 0.1542 | 0.2697 | -0.2702 | 0.2513 | -0.0191 | 0.4101 | -0.3769 | 0.2816 |
| Total polyphenols (UPLC-PDA) | -0.1461 | 0.3956 | -0.1168 | 0.6861 | 0.5345 | 0.6309 | 0.5683 | -0.0113 | 0.7035 | 0.1847 | 0.8246 | 0.7380 | 0.4610 | 0.3817 | 0.7769 |
| Colour | 0.1999 | 0.1625 | 0.1340 | -0.3462 | -0.7377 | -0.1936 | -0.1970 | -0.2497 | -0.6499 | 0.4481 | -0.0201 | 0.1894 | 0.2067 | -0.2641 | 0.0293 |
| Aroma | 0.7193 | -0.2871 | 0.6175 | -0.4715 | -0.7745 | -0.4424 | -0.3894 | -0.2616 | -0.8742 | 0.3498 | -0.4803 | -0.1319 | -0.3894 | -0.0573 | -0.4717 |
| Taste | 0.6349 | -0.4412 | 0.4441 | -0.6728 | -0.9133 | -0.6368 | -0.5920 | -0.5556 | -0.8681 | 0.1231 | -0.4159 | -0.1004 | -0.4751 | -0.3577 | -0.4554 |
| Consistency | -0.1493 | -0.0830 | -0.3196 | -0.0396 | -0.1471 | -0.0355 | -0.1319 | -0.2813 | 0.0980 | -0.0931 | 0.3844 | 0.3587 | 0.2085 | -0.3450 | 0.3400 |
| Desirability | 0.5393 | -0.2863 | 0.4167 | -0.6652 | -0.9193 | -0.5804 | -0.5306 | -0.3620 | -0.9479 | 0.2372 | -0.5012 | -0.1990 | -0.3432 | -0.3227 | -0.4877 |
| | **L*** | **a*** | **b*** | **DM[1]** | **Ashes** | **TSS[2]** | **TA[3]** | **TSS[2]/TA[3]** | **pH** | **Vitamin C** | **Fructose** | **Sorbitol** | **Glucose** | **Sucrose** | **Total sugars** |

| | **Oxalic acid** | **Citric acid** | **Tartaric acid** | **Malic acid** | **Quinic acid** | **Shikimic acid** | **Total acids** | **Sugar/ Organic acids** | **TP[4]** | **CUPRAC** | **FRAP** | **ORAC** | **DPPH•** | **ABTS•+** |
|---|---|---|---|---|---|---|---|---|---|---|---|---|---|---|
| Citric acid | 0.9989 | | | | | | | | | | | | | |
| Tartaric acid | -0.1701 | -0.2039 | | | | | | | | | | | | |
| Malic acid | 0.4810 | 0.5040 | -0.4648 | | | | | | | | | | | |
| Quinic acid | -0.1237 | -0.1041 | -0.2440 | 0.6683 | | | | | | | | | | |
| Shikimic acid | 0.8936 | 0.8986 | -0.2842 | 0.5819 | -0.1572 | | | | | | | | | |
| Total organic acids | 0.7299 | 0.7421 | -0.2721 | 0.8800 | 0.5810 | 0.6443 | | | | | | | | |
| Sugar/Organic acids | -0.3269 | -0.3207 | 0.3848 | 0.1891 | 0.1807 | -0.0379 | -0.0669 | | | | | | | |

| | | | | | | | | | | | | | | |
|---|---|---|---|---|---|---|---|---|---|---|---|---|---|---|
| TP[4] | 0.5693 | 0.5504 | 0.5996 | 0.2900 | -0.0696 | 0.5258 | 0.4747 | 0.3788 | | | | | | |
| CUPRAC | 0.4599 | 0.4342 | 0.5948 | 0.0738 | -0.2852 | 0.3504 | 0.2385 | 0.1473 | 0.8562 | | | | | |
| FRAP | 0.7590 | 0.7417 | 0.2967 | 0.4446 | -0.1120 | 0.7321 | 0.5953 | 0.1177 | 0.9006 | 0.8609 | | | | |
| ORAC | 0.6889 | 0.6914 | 0.1497 | 0.3268 | -0.3378 | 0.7925 | 0.3838 | 0.3445 | 0.7503 | 0.5985 | 0.7430 | | | |
| DPPH• | 0.7264 | 0.7034 | 0.3201 | 0.2438 | -0.3019 | 0.6396 | 0.4302 | -0.0709 | 0.8118 | 0.9160 | 0.9549 | 0.6611 | | |
| ABTS•+ | 0.7486 | 0.7280 | 0.3699 | 0.3478 | -0.1725 | 0.6763 | 0.5412 | 0.0636 | 0.9086 | 0.9035 | 0.9916 | 0.7216 | 0.9750 | |
| Total anthocyanins | -0.0399 | -0.0253 | -0.2483 | 0.3992 | 0.0058 | 0.4087 | 0.0307 | 0.6579 | 0.0829 | -0.1203 | 0.1287 | 0.3863 | -0.0398 | 0.0209 |
| Total hydroxybenzoic acids | 0.6520 | 0.6610 | -0.2826 | 0.8525 | 0.6571 | 0.5523 | 0.9788 | -0.1348 | 0.3852 | 0.1479 | 0.5163 | 0.2135 | 0.3533 | 0.4619 |
| Total hydroxycinnamic acids | -0.3460 | -0.3268 | -0.3541 | 0.5950 | 0.8845 | -0.2744 | 0.3371 | 0.2085 | -0.2644 | -0.3008 | -0.2087 | -0.4679 | -0.3353 | -0.2792 |
| Dihydrochalcones | -0.7301 | -0.7039 | -0.2445 | 0.0520 | 0.2805 | -0.4915 | -0.3711 | 0.5103 | -0.4825 | -0.4038 | -0.5566 | -0.2947 | -0.5998 | -0.6041 |
| Total flavan-3-ols | -0.1144 | -0.1010 | -0.2417 | 0.5784 | 0.3379 | 0.2901 | 0.1987 | 0.6669 | 0.0904 | -0.1186 | 0.1438 | 0.1882 | -0.0580 | 0.0263 |
| Polymeric proanthocyanidins | 0.5090 | 0.4820 | 0.6720 | 0.2037 | 0.0573 | 0.3397 | 0.4898 | 0.1662 | 0.9247 | 0.8124 | 0.8230 | 0.4734 | 0.7646 | 0.8539 |
| Total flavonols | -0.0715 | -0.0356 | -0.4303 | 0.2934 | -0.0339 | 0.2327 | -0.0422 | 0.4974 | -0.1420 | -0.2287 | -0.1488 | 0.4173 | -0.2461 | -0.2192 |
| Total polyphenols (UPLC-PDA) | 0.5733 | 0.5598 | 0.4257 | 0.5465 | 0.2302 | 0.5633 | 0.6801 | 0.3875 | 0.9354 | 0.7113 | 0.8818 | 0.6275 | 0.7272 | 0.8566 |
| Colour | -0.7722 | -0.7723 | -0.0322 | -0.0248 | 0.4360 | -0.5603 | -0.3229 | 0.3565 | -0.4480 | -0.4992 | -0.5094 | -0.6711 | -0.5907 | -0.5610 |
| Aroma | -0.6516 | -0.6709 | 0.1985 | -0.4922 | 0.1729 | -0.7465 | -0.4583 | -0.2254 | -0.4954 | -0.4230 | -0.5722 | -0.8937 | -0.5053 | -0.5366 |
| Taste | -0.7926 | -0.8174 | 0.4389 | -0.6165 | -0.0090 | -0.7999 | -0.6647 | 0.0687 | -0.3427 | -0.2177 | -0.4857 | -0.7422 | -0.4076 | -0.4455 |
| Consistency | -0.0394 | -0.0674 | 0.5401 | 0.0538 | -0.0884 | 0.1754 | -0.0294 | 0.5587 | 0.5532 | 0.4430 | 0.4938 | 0.2368 | 0.4037 | 0.4619 |
| Desirability | -0.8706 | -0.8806 | 0.0955 | -0.5136 | 0.1288 | -0.8357 | -0.6505 | -0.0192 | -0.6508 | -0.5416 | -0.7380 | -0.8912 | -0.6763 | -0.7218 |
| | **Oxalic acid** | **Citric acid** | **Tartaric acid** | **Malic acid** | **Quinic acid** | **Shikimic acid** | **Total acids** | **Sugar/ Organic acids** | **TP[4]** | **CUPRAC** | **FRAP** | **ORAC** | **DPPH•** | **ABTS•+** |

| | **Total anthocyanins** | **Total hydroxybenzoic acids** | **Total hydroxycinnamic acids** | **Dihydrochalcones** | **Total flavan-3-ols** | **Polymeric proanthocyanidins** | **Total flavonols** | **Total Polyphenols (UPLC-PDA)** | **Colour** | **Aroma** | **Taste** | **Consistency** |
|---|---|---|---|---|---|---|---|---|---|---|---|---|
| Total hydroxybenzoic acids | -0.0182 | | | | | | | | | | | |
| Total hydroxycinnamic acids | 0.1698 | 0.4196 | | | | | | | | | | |
| Dihydrochalcones | 0.4118 | -0.3918 | 0.5591 | | | | | | | | | |
| Total flavan-3-ols | 0.9217 | 0.1984 | 0.5007 | 0.4589 | | | | | | | | |
| Polymeric proanthocyanidins | -0.2063 | 0.4643 | -0.1810 | -0.6260 | -0.1047 | | | | | | | |
| Total flavonols | 0.6685 | -0.1801 | 0.0902 | 0.6503 | 0.4969 | -0.4887 | | | | | | |

| | | | | | | | | | | | | |
|---|---|---|---|---|---|---|---|---|---|---|---|---|
| Total polyphenols (UPLC-PDA) | 0.1790 | 0.6325 | 0.0259 | -0.4371 | 0.2902 | 0.8993 | -0.1694 | | | | | |
| Colour | 0.3415 | -0.1834 | 0.6391 | 0.5682 | 0.5431 | -0.3572 | -0.0479 | -0.2526 | | | | |
| Aroma | -0.3593 | -0.2725 | 0.2343 | 0.0453 | -0.1886 | -0.1942 | -0.6386 | -0.4125 | 0.6864 | | | |
| Taste | -0.1881 | -0.5191 | 0.1438 | 0.2055 | -0.0663 | -0.1299 | -0.4987 | -0.3415 | 0.7280 | 0.9117 | | |
| Consistency | 0.5000 | 0.0025 | -0.0118 | -0.1253 | 0.5612 | 0.5135 | -0.2005 | 0.5914 | 0.3684 | 0.1079 | 0.3386 | |
| Desirability | -0.1192 | -0.4938 | 0.3150 | 0.3976 | 0.0056 | -0.4545 | -0.3132 | -0.5873 | 0.8257 | 0.9220 | 0.9261 | 0.0905 |
| | **Total anthocyanins** | **Total hydroxybenzoic acids** | **Total hydroxycinnamic acids** | **Dihydrochal-cones** | **Total flavan-3-ols** | **Polymeric proanthocyanidins** | **Total flavonols** | **Total Polyphenols (UPLC-PDA)** | **Colour** | **Aroma** | **Taste** | **Consistency** |

Legend

| | | | |
|---|---|---|---|
| DM[1] | Dry matter | | |
| TSS[2] | Total soluble solids | | significant at $p \leq 0.05$ |
| TA[3] | Total acidity | | significant at $p \leq 0.01$ |
| TP[4] | Total phenols by Folin-Chiocalteu's assay | | |

**Table S2**. Quantification of phenolic compounds by UPLC-PDA method (mg/100 g fw).

| Code | Compound | $R_t$ (min) | $λ_{max}$ (nm | MS [M-H]$^-$ (*m/z*)* | MS/MS [M-H]$^-$ (*m/z*) | Juice composition (mg/100 g fw) | | | | | | |
|---|---|---|---|---|---|---|---|---|---|---|---|---|
| | | | | | | AJ | AJ+S01 | AJ+S05 | AJ+M5 | AJ+F5 | AJ+P5 | AJ+C5 |
| Anthocyanins | | | | | | | | | | | | |
| A1 | Delphinidin-3,5-*O*-diglucoside | 3.040 | 518 | 627.2785$^+$ | 465.1909/303.1100 | nd | 0.17±0.01b | 1.77±0.02a | nd | nd | nd | nd |
| A2 | Delphinidin-3-*O*-glucoside | 3.856 | 515 | 465.1953$^+$ | 303.1100 | nd | nd | 0.37±0.04b | 10.55±0.31a | nd | nd | nd |
| A3 | Delphinidin-pentoside | 4.133 | 520 | 435.1818$^+$ | 303.1100 | nd | nd | nd | 0.23±0.02a | nd | nd | nd |
| A4 | Cyanidin-3-*O*-galactoside | 4.135 | 515 | 449.1959$^+$ | 287.1116 | nd | nd | nd | nd | nd | nd | 1.04±0.11a |
| A5 | Cyanidin-3-*O*-glucoside | 4.397 | 520 | 449.1959$^+$ | 287.1116 | nd | nd | nd | 3.32±0.26b | 3.69±0.36a | nd | nd |
| A6 | Cyanidin-3-*O*-arabinoside | 4.629 | 515 | 419.1797$^+$ | 287.1116 | nd | nd | nd | nd | nd | nd | 0.04±0.00a |
| A7 | Petunidin-3-*O*-glucoside | 4.648 | 525 | 479.2150$^+$ | 317.1295 | nd | nd | nd | 0.83±0.11a | nd | nd | nd |
| A8 | Peonidin-3-*O*-glucoside | 5.156 | 525 | 463.2164$^+$ | 301.1330 | nd | nd | nd | 0.04±0.00a | nd | nd | nd |
| A9 | Malvidin-3-*O*-glucoside | 5.391 | 519 | 493.2339$^+$ | 331.1495 | nd | nd | nd | 17.05±0.24a | nd | nd | nd |
| Total | | | | | | **nd** | **0.17±0.01e** | **2.14±0.03c** | **32.02±0.15a** | **3.69±0.36b** | **nd** | **1.08±0.05d** |
| Hydroxybenzoic acids | | | | | | | | | | | | |
| B1 | Gallic acid glucoside I | 1.156 | 280 | 331.1266 | 271.1605/169.1417 | nd | nd | nd | nd | nd | nd | 1.03±0.02a |
| B2 | Galloyl glucoside I | 1.274 | 277 | 331.1334 | 169.0417 | nd | nd | nd | nd | nd | nd | 2.06±0.02a |
| B3 | Galloyl HHDP-glucose I | 1.325 | 271 | 633,1283 | 481.0828/301.0667 | nd | nd | nd | 2.52±0.03a | nd | nd | nd |
| B4 | Galloyl glucoside II | 1.331 | 270 | 331,0639 | 169.0117 | nd | nd | nd | nd | nd | 1.10±0.01a | nd |
| B5 | Gallic acid glucoside II | 1.366 | 270 | 331.1334 | 271.1990/169.1417 | nd | nd | nd | nd | nd | nd | 0.54±0.01a |
| B6 | 3-*O*-Galloylquinic acid (Theogallin) | 1.551 | 273 | 343.0742 | 191.1410 | nd | nd | nd | nd | nd | nd | 23.10±0.74a |
| B7 | Galloyl HHDP-glucose II | 1.591 | 272 | 633,1283 | 481.0828/301.0667 | nd | nd | nd | 2.43±0.06a | nd | nd | nd |
| B8 | Galloyl glucoside III | 1.627 | 273 | 331,0639 | 169.0117 | nd | nd | nd | nd | nd | 1.70±0.12a | nd |
| B9 | Digalloyl-HHDP-glucose | 1.892 | 280 | 785,1292 | 633.0180/481.0828/301.0631 | nd | nd | nd | 0.39±0.02a | nd | nd | nd |
| B10 | Gallic acid 4-*O*-β-D-glucopyranoside | 2.080 | 320 | 331.1334 | 169.0417 | nd | nd | nd | nd | nd | nd | 0.03±0.00a |
| B11 | Castalagin | 2.084 | 280 | 933.1019 | 785.1813/481.0917/301.1057 | nd | nd | nd | nd | 7.87±0.52a | nd | nd |
| B12 | Galloyl shikimic acid | 2.314 | 272 | 325.0878 | 169.0417/125.4180 | nd | nd | nd | nd | nd | nd | 0.70±0.09a |
| B13 | Casuarin | 2.420 | 374 | 783.1445 | 481.0421/301.0667 | nd | nd | nd | nd | 1.15±0.03a | nd | nd |

| | | | | | | | | | | | | |
|---|---|---|---|---|---|---|---|---|---|---|---|---|
| B14 | Ellagitannin I | 2.435 | 280 | 933.1457 | 633.0283/481.0783/301.0667 | nd | nd | nd | 0.13±0.01a | nd | nd | nd |
| B15 | Digalloylquinic acid I | 2.755 | 273 | 495.1837 | 343.1255/191.3072 | nd | nd | nd | nd | nd | nd | 0.21±0.03a |
| B16 | Ellagitannin II | 2.829 | 270 | 933.1114 | 781.0445/633.7131/301.1057 | nd | nd | nd | nd | 9.34±0.12a | nd | nd |
| B17 | Ellagitannin III | 3.035 | 275 | 783.0645 | 481.0186/301.1021 | nd | nd | nd | 1.50±0.21a | nd | nd | nd |
| B18 | Digalloylquinic acid II | 3.210 | 276 | 495.0435 | 343.1158/191.3100 | nd | nd | nd | nd | nd | nd | 0.26±0.02a |
| B19 | Ellagitannin IV | 3.331 | 280 | 783.0759 | 481.0917/301.0667 | nd | nd | nd | nd | 0.64±0.03a | nd | nd |
| B20 | Nilocitin | 3.653 | 270 | 481.0938 | 301.0667/257.1438 | nd | nd | nd | nd | 0.26±0.02a | nd | nd |
| B21 | Digalloyl shikimic acid I | 4.129 | 278 | 477.0493 | 325.0808/169.0417 | nd | nd | nd | nd | nd | nd | 0.98±0.08a |
| B22 | Casuarinin | 4.391 | 278 | 935.0146 | 765.1799/545.1230 | nd | nd | nd | nd | 3.56±0.23a | nd | nd |
| B23 | Digalloyl shikimic acid II | 4.618 | 275 | 477.0493 | 325.0806/169.0417 | nd | nd | nd | nd | nd | nd | 0.18±0.02a |
| B24 | Strictinin ellagitannin | 4.700 | 275 | 633.0900 | 463.1637/301.0667/275.1263 | nd | nd | nd | nd | nd | nd | 0.21±0.01a |
| B25 | Ellagic acid arabinoside | 5.703 | 359 | 433.0224 | 301.0631 | nd | nd | nd | nd | 0.87±0.06a | nd | nd |
| B26 | Ellagic acid xyloside | 5.885 | 361 | 433.0735 | 301.0631 | nd | nd | nd | nd | 0.52±0.03a | nd | nd |
| B27 | Ellagic acid | 6.028 | 366 | 300.0631 | | nd | nd | nd | nd | 3.04±0.13a | nd | nd |
| B28 | Methyl ellagic acid | 8.128 | 360 | 394.0062 | 315.1020/301.0631 | nd | nd | nd | nd | 1.11±0.24a | nd | nd |
| Total | | | | | | **nd** | **nd** | **nd** | **6.97±0.13c** | **28.36±0.15b** | **2.80±0.05d** | **29.28±0.11a** |
| Hydroxycinnamic acids | | | | | | | | | | | | |
| C1 | Neochlorogenic acid | 3.489 | 317 | 353.1287 | 191.3100/136.0212 | 0.33±0.03de | 0.55±0.09a | 0.27±0.02e | 0.46±0.05b | 0.45±0.02bc | 0.37±0.01cd | 0.42±0.02bc |
| C2 | Chlorogenic acid | 3.723 | 323 | 353.0838 | 191.0534 | 5.00±0.11c | 5.43±0.06b | 5.42±0.22b | 5.46±0.32b | 4.81±0.16c | 5.10±0.21bc | 6.62±0.21a |
| C3 | Caffeic acid | 4.036 | 320 | 311.0807 | 179.1098 | 0.20±0.02d | 0.21±0.02cd | 0.21±0.01cd | 0.27±0.03b | 0.33±0.01a | 0.23±0.01cd | 0.24±0.03bc |
| C4 | *p*-Coumaric acid | 4.463 | 323 | 163.0349 | | 0.13±0.00c | 0.14±0.01bc | 0.15±0.01b | 0.28±0.02a | 0.10±0.02d | 0.13±0.01c | 0.15±0.00bc |
| C5 | *p*-Coumaroyloquinic acid | 4.904 | 310 | 337.0912 | 163.1014 | 0.92±0.11a | 0.99±0.03a | 1.01±0.00a | 0.97±0.11a | 0.93±0.08a | 0.93±0.11a | 0.99±0.03a |
| Total | | | | | | **6.58±0.05e** | **7.32±0.03b** | **7.06±0.02c** | **7.44±0.04b** | **6.62±0.10de** | **6.76±0.13d** | **8.42±0.11a** |
| Dihydrochalcones | | | | | | | | | | | | |
| D1 | Phloretin-2'-*O*-xyloglucoside | 7.433 | 280 | 567.1703 | 273.0757 | 2.08±0.42bc | 2.22±0.04ab | 2.43±0.10a | 2.27±0.01ab | 1.78±0.11c | 2.10±0.04ab | 2.23±0.06ab |
| D2 | Phloretin-2'-*O*-glucoside (Phloridzin) | 8.186 | 280 | 435.1332 | 273.0733 | 2.53±0.13b | 2.74±0.17ab | 2.76±0.05ab | 2.89±0.12a | 2.54±0.08b | 2.61±0.11b | 2.72±0.11ab |
| Total | | | | | | **4.61±0.10c** | **4.96±0.08b** | **5.19±0.11a** | **5.16±0.09a** | **4.32±0.10d** | **4.71±0.12c** | **4.95±0.12b** |
| Flavan-3-ols | | | | | | | | | | | | |
| E1 | Procyanidin B1 | 3.256 | 280 | 577.1293 | 289.0708 | 1.49±0.06c | 1.61±0.10c | 1.13±0.11d | 11.25±0.23a | 1.09±0.11d | 1.60±0.06c | 2.00±0.02b |

| | | | | | | | | | | | | |
|---|---|---|---|---|---|---|---|---|---|---|---|---|
| E2 | Procyanidin B3 | 3.562 | 277 | 577.1055 | 289.2014/245.2391 | nd | nd | nd | nd | nd | nd | 0.06±0.00a |
| E3 | (+)-Catechin | 3.669 | 280 | 289.0673 | 245.0780 | 1.48±0.11e | 1.79±0.05d | 1.44±0.14e | 2.18±0.22c | 4.17±0.32b | 1.92±0.06cd | 5.72±0.14a |
| E4 | (-)-Epiatechin | 4.143 | 280 | 289.0673 | 245.0780 | 5.98±0.04d | 7.20±0.22b | 6.60±0.03c | 7.20±0.05b | 6.07±0.12d | 6.83±0.11c | 7.94±0.21a |
| E5 | Procyanidin B2 | 4.631 | 280 | 577.1293 | 289.0708 | 1.65±0.11bc | 1.79±0.06b | 1.80±0.02b | 6.43±0.11a | 1.45±0.12d | 1.76±0.04bc | 1.62±0.12c |
| E6 | Procyanidin C1 | 4.956 | 280 | 866.1908 | 577.1188/289.0708 | 0.40±0.01c | 0.79±0.10a | 0.61±0.03b | 0.74±0.04a | 0.44±0.02c | 0.80±0.03a | 0.62±0.04b |
| Total | | | | | | **11.00±0.97d** | **13.18±0.79c** | **11.58±0.60d** | **27.80±1.24a** | **13.22±0.88c** | **12.91±0.67c** | **17.96±1.12b** |
| PP# | Polymeric proanthocyanidins | | | | | 108.11±1.25e | 109.15±2.56e | 88.69±3.64f | 118.40±2.54d | 209.99±7.45b | 242.68±3.56a | 160.66±4.56c |
| DP | Degree of polymerisation | | | | | 3.53 | 3.64 | 3.28 | 3.66 | 4.78 | 4.10 | 4.11 |
| Flavonols | | | | | | | | | | | | |
| F1 | Kaempferol-3-*O*-sophoroside-7-*O*-glucoside | 3.546 | 346 | 771.0181 | 609.0240/2851257 | nd | 0.39±0.02b | 2.06±0.11a | nd | nd | nd | nd |
| F2 | Quercetin-3,7-*O*-digalactoside | 4.519 | 349 | 625.0223 | 463.0290/301.0951 | nd | nd | 0.06±0.00a | nd | nd | nd | nd |
| F3 | Kaempferol-3,7-*O*-diglucoside | 4.636 | 345 | 609.0341 | 447.0543/285.1292 | nd | 0.04±0.00b | 0.15±0.01a | nd | nd | nd | nd |
| F4 | Isorhamnetin-3,7-*O*-digalacto-side | 4.747 | 350 | 639.0883 | 447.0327/315.0605 | nd | 0.24±0.03b | 0.41±0.05a | nd | nd | nd | nd |
| F5 | Quercetin derivative I | 4.766 | 359 | 633.0900 | 463.0633/301.0667 | nd | nd | nd | nd | 0.09±0.00b | nd | 0.21±0.02a |
| F6 | Myricetin galactoside-gallate | 4.995 | 360 | 631.1107 | 479.1073/317.1114 | nd | nd | nd | 1.75±0.10a | nd | nd | nd |
| F7 | Quercetin-3,7-*O*-diglucoside | 5.356 | 352 | 625.1044 | 463.0333/301.0924 | nd | 0.35±0.02b | 2.26±0.22a | nd | 0.09±0.02c | nd | nd |
| F8 | Myricetin-3-*O*-galactoside | 5.460 | 356 | 479.0610 | 317.1114 | nd | nd | nd | 9.85±0.54a | nd | nd | 0.33±0.02b |
| F9 | Myricetin-3-*O*-glucoside | 5.534 | 356 | 479.0162 | 317.1114 | nd | nd | nd | 0.50±0.08a | nd | nd | 0.25±0.03b |
| F10 | Isorhametin-3,7-*O*-diglucoside | 5.709 | 343 | 639.1035 | 477.1314/315.0948 | nd | 0.17±0.00b | 1.18±0.02a | nd | nd | nd | nd |
| F11 | Quercetin galloylhexose | 5.816 | 360 | 615.1291 | 463.0950/301.1092 | nd | nd | nd | nd | nd | nd | 0.08±0.00a |
| F12 | Kaempferol-3-*O*-sophoroside | 5.978 | 358 | 609.0240 | 285.1226 | nd | 2.13±0.02b | 11.84±0.35a | nd | nd | nd | nd |
| F13 | Myricetin-3-*O*-arabinoside | 6.027 | 364 | 449.0362 | 317.0678 | nd | nd | nd | 1.18±0.01a | nd | nd | nd |
| F14 | Quercetin derivative II | 6.053 | 366 | 633.1003 | 463.0861/301.1021 | nd | nd | nd | nd | nd | nd | 0.27±0.01a |
| F15 | Isorhamnetin-3-*O*-sophoroside | 6.158 | 352 | 639.0983 | 315.0948 | nd | nd | 0.20±0.01a | nd | nd | nd | nd |
| F16 | Myricetin-3-*O*-xyloside | 6.161 | 350 | 449.1883 | 317.1114 | nd | nd | nd | nd | nd | nd | 0.03±0.00a |
| F17 | Myricerin-3-*O*-rhamnoside | 6.239 | 347 | 463.0861 | 317.1114 | nd | nd | nd | 5.88±0.22a | nd | nd | 0.02±0.00b |
| F18 | Quercetin-3-*O*-galactoside | 6.345 | 348 | 463.0861 | 301.0994 | 0.57±0.07d | 0.67±0.12cd | 0.57±0.03d | 1.05±0.02b | 1.28±0.04a | 0.66±0.02cd | 0.75±0.11c |
| F19 | Quercetin-3-*O*-glucoside | 6.494 | 350 | 463.1774 | 301.1447 | 0.10±0.00f | 0.21±0.02c | 0.40±0.01a | 0.21±0.02bc | 0.23±0.01b | 0.14±0.00e | 0.17±0.01d |
| F20 | Kaempferol-3-*O*-galactoside | 6.640 | 347 | 447.0629 | 285.1326 | nd | nd | nd | nd | 0.53±0.03a | nd | nd |

| | | | | | | | | | | | | |
|---|---|---|---|---|---|---|---|---|---|---|---|---|
| F21 | Isorhamnetin-3-*O*-rutinoside | 6.697 | 352 | 623.1223 | 315.0871 | nd | 0.22±0.02b | 1.32±0.06a | nd | nd | nd | nd |
| F22 | Quercetin-3-*O*-arabinoside | 6.765 | 350 | 433.0160 | 301.1421 | 0.14±0.00b | 0.18±0.03b | 0.16±0.00b | 0.17±0.04b | 0.27±0.03a | 0.15±0.01b | 0.15±0.01b |
| F23 | Quercetin-pentoside | 6.957 | 360 | 433.0565 | 301.0959 | nd | nd | nd | nd | 0.39±0.02a | nd | nd |
| F24 | Quercetin-3-*O*-xyloside | 7.063 | 350 | 433.0160 | 301.1421 | 0.32±0.04c | 0.41±0.03b | 0.36±0.04bc | 0.42±0.01b | 0.68±0.10a | 0.41±0.01b | 0.74±0.02a |
| F25 | Quercetin-3-*O*-rhamnoside | 7.325 | 348 | 447.0148 | 301.1447 | 0.50±0.03f | 0.84±0.05d | 1.52±0.11a | 1.27±0.12b | 0.53±0.03f | 0.68±0.04e | 1.07±0.06c |
| F26 | Isorhanetin-3-*O*-glucoside | 7.539 | 352 | 477.1314 | 315.0871 | nd | nd | 0.23±0.02a | nd | nd | nd | nd |
| F27 | Kaempferol-hexoside | 7.568 | 350 | 447.1543 | 285.1326 | nd | nd | nd | nd | 0.21±0.01a | nd | nd |
| F28 | Myricetin | 7.683 | 367 | 317.1114 | | nd | nd | nd | 0.33±0.03a | nd | nd | nd |
| F29 | Quercetin | 8.774 | 360 | 301.1065 | | nd | nd | nd | nd | 0.05±0.00a | nd | nd |
| F30 | Kaempferol | 9.435 | 360 | 285.1706 | | nd | nd | nd | nd | 3.78±0.12a | nd | nd |
| Total | | | | | | **1.63±0.06f** | **5.85±0.10c** | **22.72±0.13a** | **22.61±0.11a** | **8.12±0.05b** | **2.04±0.03e** | **4.07±0.06d** |
| Total phenolic compounds (including polymeric proanthocyanidins) | | | | | | **131.93±3.45d** | **140.64±5.12c** | **137.37±3.44cd** | **220.40±8.21b** | **274.32±4.22a** | **271.90±3.03a** | **226.41±4.21b** |

Data are given as mean ± standard deviation (n=3). nd: not detected. tr: traces. Mean values within a column with different letters (a-f) are significantly different (homogenous groups) at $p \leq 0.05$. * $[M+H]^+$ (m/z) for anthocyanins were obtained in the positive ion mode. [#] Quantitative data of polymeric proanthocyanidins were obtained using the phloroglucinol method.

**Table S3.** Consumer evaluation (5° hedonic scale).

| Juice composition | Qualitative discriminate | | | | |
|---|---|---|---|---|---|
| | Colour | Aroma | Taste | Consistency | Desirability |
| AJ | 3.40±0.70a | 4.60±0.70a | 4.30±0.67a | 2.90±1.20a | 4.50±0.53a |
| AJ+S01 | 3.25±0.86ab | 3.85±0.58ab | 4.00±0.67a | 2.90±1.20a | 4.10±0.99a |
| AJ+S05 | 2.50±0.97ab | 2.70±0.67bc | 2.95±0.76ab | 2.80±1.32a | 3.20±0.79ab |
| AJ+M5 | 3.85±1.16a | 2.90±1.10bc | 3.40±1.17ab | 3.00±1.25a | 3.50±1.27ab |
| AJ+F5 | 1.60±0.84b | 2.15±1.06c | 2.15±1.06b | 2.90±1.29a | 1.85±1.11b |
| AJ+P5 | 2.95±0.83ab | 3.60±0.52abc | 4.15±0.47a | 3.00±1.15a | 3.55±1.07ab |
| AJ+C5 | 3.90±0.74a | 4.10±0.74ab | 3.75±0.86a | 2.90±0.99a | 4.00±1.33a |

Data are given as mean ± standard deviation (n=3). Mean values within a column with different letters (a-c) are significantly different (homogenous groups) at $p \leq 0.05$.

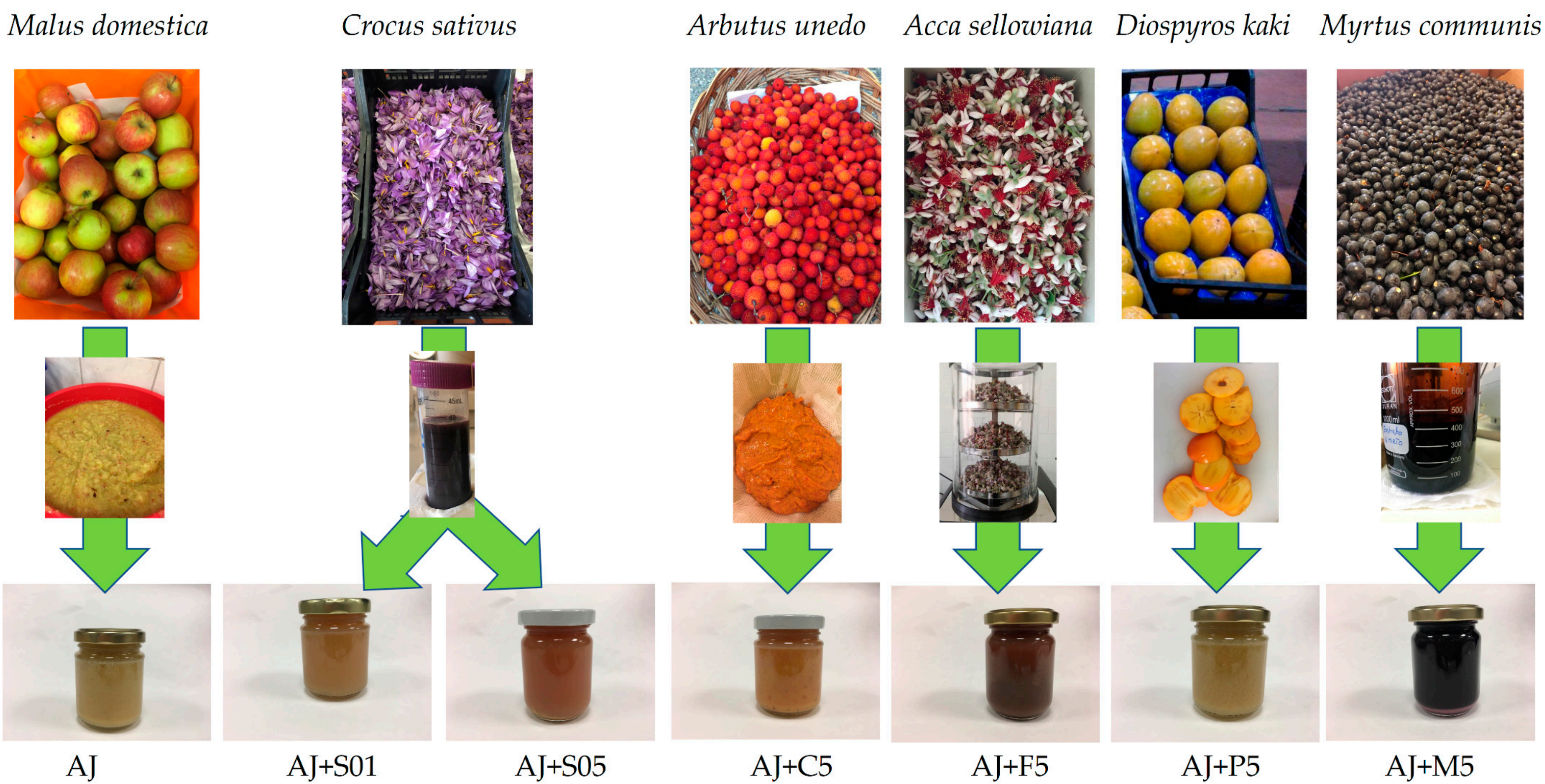


**Figure S1**. Plant material used for the investigation, semi-finished products and the obtained final smoothies.
